# Relationship between the Well-Being of Elderly Men and Cohabiting with Women Who Have Had Experience as a Health Promotion Volunteer in Japan: A Cross-Sectional Study

**DOI:** 10.3390/ijerph16010065

**Published:** 2018-12-27

**Authors:** Haruhiko Imamura, Hideki Nakamura, Yuji Nishiwaki

**Affiliations:** 1 Department of Environmental and Occupational Health, School of Medicine, Toho University, Tokyo 1438540, Japan; yuuji.nishiwaki@med.toho-u.ac.jp; 2 Health and Welfare Department, Suzaka City Hall, Suzaka City, Nagano 3828511, Japan; hideki.nakamura@city.suzaka.nagano.jp

**Keywords:** health promotion volunteer, well-being, functional capacity, depressive symptoms, family

## Abstract

In Japan, there are traditionally many health promotion volunteer activities. However, the effects these activities have on the volunteers’ families are not clear. This study examined whether the well-being of Japanese elderly men was affected by cohabiting with women who have had experience as a health promotion volunteer. The study area was Suzaka City, where more than 7500 women have been elected and served as health promotion volunteers for over 60 years. A cross-sectional survey targeting all residents aged 65 years or over was conducted in 2014 using a self-administered questionnaire and 10,758 (77.7%) residents participated. Of those, married men who lived with married women were analyzed (*n* = 2370). Functional capacity and depressive symptoms were analyzed as outcomes respectively. Of the 2370 men, 1434 (60.5%) lived with women who had experience as a health promotion volunteer in the past. Modified Poisson regression analysis adjusting for covariates showed that living with women who had this experience was inversely associated with depressive symptoms (adjusted Prevalence Ratio; 0.84, 95% Confidence Interval; 0.73–0.97), but not with low functional capacity. These results suggest that living with women who had the experience as health promotion volunteer might affect depressive symptoms of elderly men.

## 1. Introduction

There are a variety of health promotion volunteer organizations (a type of community organization) and activities in Japan, which operate under the support of a municipal public health nurse. Further, health promotion volunteering has played an important role in public health in Japan. For example, the Nagano prefecture, which is located in central Japan and had the highest average life expectancy for both men and women in 2010 [1], has an historical background of health promotion volunteers being engaged in public health activities throughout the prefecture [2]. There have been more than 10,000 health promotion volunteers annually since 1979 in the Nagano prefecture [3]. These volunteers have been engaged in various activities, such as improvement of hygiene and nutrition, promotion of blood pressure checks, and prevention of stroke (conducting room temperature surveys and salt level checks) in their families and neighborhoods [2].

Several studies have suggested that experience as a health promotion volunteer is associated with the volunteer’s own health behaviors, health literacy, and health conditions [4,5,6,7]. For example, elderly women who have experience as a health promotion volunteer have a better condition of functional capacity and Activities of Daily Living (ADL) [6], and their inpatient medical cost was lower than those who had not had volunteering experience [7]. However, there has been little evidence showing the effects of the volunteering on the health of the volunteers’ families. It is possible that the health promotion volunteers’ experience could affect their family’s health, because the role of health promotion volunteer is to promote not only their own health but also, more generally, the health of their family and neighborhood. Furthermore, there are many studies which suggest that marital and cohabitation statuses are important factors for health status [8,9,10,11,12,13,14]. In addition, previous studies have indicated that there is concordance in health status such as frailty, depressive symptoms, physical health, and health behavior within married couples [15,16].

The purpose of the current study is to examine whether the well-being of elderly men is affected by cohabiting with women who have had experience as a health promotion volunteer. The study was conducted using cross-sectional survey data in a Japanese suburban city, where more than 7500 women have been elected and served as health promotion volunteers for over 60 years.

## 2. Materials and Methods 

### 2.1. Study Area

The study area was Suzaka City, Nagano prefecture, Japan, which is located adjacent to the prefectural capital (as of February 2014, 14,792 (28.4%) of its population of 52,176 were aged 65 years or older). Suzaka City’s health promotion volunteer organization (“Hoken-hodouin” organization) has the oldest history in Nagano prefecture and was originally established in 1945 in the former Takaho village, based on the idea of using women residents and the village’s public health nurses collaboratively to improve hygiene and prevent infectious disease [17]. Later, five neighboring municipalities merged to become Suzaka City, and activities as the current organization began in 1958. All health promotion volunteers have been women and have served 2-year terms each. This principle aims to have as many women as possible acquire health knowledge and experience health promotion activities [17]. Health promotion volunteers are elected from each of the 67 wards in the city [18]. The elections are mainly conducted using a rotation system among neighborhood associations. For these reasons, any woman in Suzaka City can be elected, regardless of health consciousness or health knowledge level [3]. In 2015, there were 271 health promotion volunteers (average age: 60.9). In recent years, in each term, there were approximately the same number of health promotion volunteers. The cumulative total number of health promotion volunteers is over 7500 (these information were obtained from Suzaka City Hall). Activities are performed based on wards or the 10 blocks that each ward consists of [3], and leadership roles such as “leader” and “subleader” are assigned within each block. In the 2-year term, health promotion volunteers take block-based health learning programs with the support of Suzaka City’s public health nurses. For example, in 2015, a total of 2054 health promotion volunteers participated in a total of 90 programs (information provided by Suzaka City Hall). Additionally, the volunteers have experience with health promotion activities for their family and local community, such as recommendations for health examination, hosting “child-rearing salons”, supporting health education classes, and promotion of the physical exercise [3,18]. Although elections are conducted using a rotation system, activity satisfaction is generally high and there are many cases of activities continuing after the 2-year term (e.g., alumni meetings and engaging in new volunteer activities by utilizing the experience as health promotion volunteer) [3,17].

### 2.2. Study Population

A self-administered cross-sectional questionnaire survey on health and daily life, including questions about women’s experience as health promotion volunteers, was conducted in Suzaka City. The target population were those aged 65 or older as of 1 February 2014. However, those who were facility residents, hospital inpatients, or who met degree 4 or more (out of 5) in their long-term care insurance service were excluded from the survey. Long-term care insurance services comprise the national health service in Japan, for which every Japanese person aged 65 and older is eligible [19]. Degree 4 of long-term care insurance service means that the individual has severe dementia and needs full assistance with daily life. We sent the questionnaire to 13,846 individuals in February 2014, and received responses from 10,758 individuals (a response rate of 77.7%). Next, from the database of administrative information of Suzaka City, we acquired household information and merged this with the questionnaire responses using anonymized IDs. This household information enabled us to analyze responses from same household.

**Figure 1:**
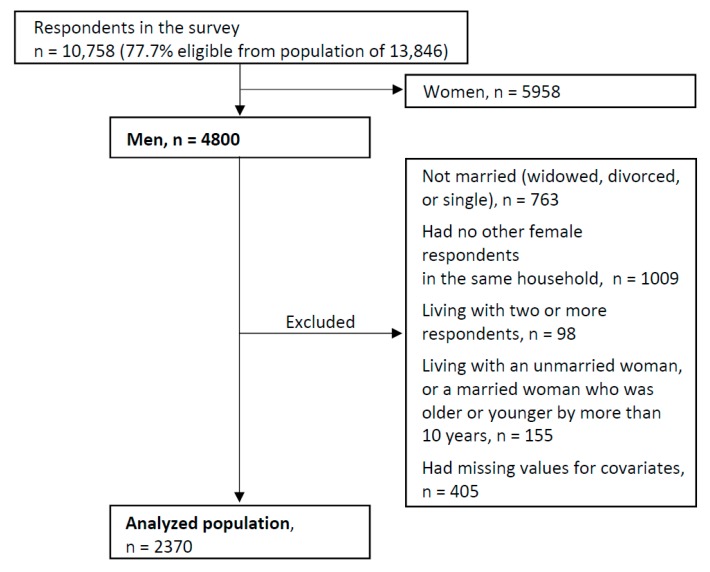
Study population.

Of those participants, 4800 (44.6%) were men. In this study, we intended to analyze men from married couples. However, in this dataset, we could not identify the respondents’ marital relationship who was married with whom, even if we could identify the respondents’ marital status and household information. Therefore, we analyzed the data from married men who lived with married women who were close in age (plus or minus 10 years from the man’s age), suggesting that they were the man’s spouse. Of the participating men, we excluded those who were not married (*n* = 763), who had no other women respondent in the same household (*n* = 1009), who were living with two or more respondents, (*n* = 98), who were living with unmarried woman or married woman who were older or younger than the men by more than 10 years (*n* = 155), and had missing values for covariates (*n* = 405). After these exclusions, we were left with 2370 whose data were analyzed in the study (Figure 1). The study was conducted in accordance with the Declaration of Helsinki, and the protocol was approved by the Ethics Committee of Faculty of Medicine, Toho University (No. 25103).

### 2.3. Exposure Measurement

The exposure in this study was the presence of cohabiting women who had experience as a health promotion volunteer. From the questions asked of the women about their health promotion volunteer experience (including those currently volunteering), the responses of women from the same household were dichotomized into “not experienced” or “experienced”. “Experienced” meant that the cohabiting women would have served as a health promotion volunteer for two years. In addition, within “experienced”, the following three subcategories were also set: years since experience (age now minus age at experience; “0–19 years”, “20–39 years”, and “40 years or more”), leadership role (whether they had experience in a leadership role or not; “no” or “yes”), and satisfaction with the experience (responses to the question “Do you think that it was good to experience health promotion volunteer?”; “low; disagree/somewhat disagree/neither agree nor disagree”, “medium; agree”, and “high; strongly agree”).

### 2.4. Outcome Measurement

We set two outcomes which represented well-being in elderly people: functional capacity and depressive symptoms. Functional capacity was measured using the Tokyo Metropolitan Institute of Gerontology Index of Competence (TMIG-IC), which measures higher-level competence, such as instrumental self-maintenance, intellectual activity, and social role in elderly people using 13 items [20]. The cutoff value was 10 points out of 13 points, with 10 points or less being treated as “low”. This cutoff value was set based on the average value of Japanese people aged over 65 [21]. Depressive symptoms were measured using the 5-item Geriatric Depression Scale (GDS5), which was developed as a short form of the 30- or 15-item GDS for the purposes of screening for depression in elderly people more conveniently and effectively [22]. The cutoff value was 2 out of 5 points, with 2 points or more being treated as “have depressive symptoms”. The validity of the GDS5 and the cutoff value has been previously verified: a sensitivity of 0.94 and a specificity of 0.81 for clinical diagnosis of depression. GDS5 also had good interrater reliability (kappa index = 0.88) and test–retest reliability (kappa index = 0.84) [23].

### 2.5. Covariates

In addition to age, socioeconomic status, health condition, and health-related behavior of the study population were considered as covariates that might be related to the well-being of elderly people. Educational attainment and equivalent household income (calculated as household annual income, divided by the square root of the number of people per household) were selected to represent socioeconomic status; history of major diseases was selected to represent health condition; and exercise habits, consciousness of healthy eating habits, current drinking, and current smoking were selected to represent health-related behaviors. Equivalent household income was obtained from the Suzaka City database of administrative information, and the other covariates were from the questionnaire. History of major diseases was defined as having any one of the following diseases known to be causes of death or disability in older adults; stroke, myocardial infarction/angina, diabetes, Parkinson’s disease, femoral neck fracture, and cancer. Age was a continuous variable, and the other covariates were divided into two or four categories each: educational attainment (“≥10 years” and “<10 years”); equivalent household income (four quartiles of all respondents); history of major diseases (“no” and “yes”); exercise habits (“one hour or more per week” and “less than one hour per week”); consciousness of healthy eating habits (“conscious” and “not conscious”); current drinking (“no” and “yes”); and current smoking (“no” and “yes”).

### 2.6. Statistical Analysis

Prevalence ratios (PRs) of low functional capacity and depressive symptoms were estimated respectively using a modified Poisson regression analysis to analyze whether the cohabiting women’s experience as a health promotion volunteer was associated with the well-being of the study population. The modified Poisson regression analysis rectify variance overestimation that occur when Poisson regression is applied to binary data and estimate effect measure directly by using a robust error variance [24]. The reference category was “not experienced”. First, the association of each exposure variable with outcomes was assessed in the model, adjusting for age (Model 1). Next, educational attainment and equivalent household income were added to Model 1 (Model 2). Finally, history of major diseases, exercise habits, consciousness of healthy eating habits, current drinking, and current smoking were added to Model 2 (Model 3). The statistical significance level was set at *p* < 0.05. All analyses were performed with STATA, version 14.0 (STATA Corporation, College Station, TX, USA).

## 3. Results

The mean age of the study population (*n* = 2370) was 74.8 years (standard deviation; 5.8). Table 1 shows the characteristics of the study population stratified by exposure variable. Of these, 60.5% (*n* = 1434) lived with women who had experience as a health promotion volunteer. These respondents were older, more educated, and had a higher equivalent household income than those who did not live with women who had experience.

Table 2 shows the association between the cohabiting women’s experience as a health promotion volunteer and functional capacity (TMIG-IC) in the study population (elderly men from the same household). Among those analyzed, 18.4% (414/2247) had low functional capacity. The prevalence of low functional capacity in the “experienced” group was comparable with that in the “not experienced” group (18.3% vs. 18.6%). In the modified Poisson regression analyses, the adjusted PR in Model 3 was 1.02 (95% CI; 0.87–1.21). With regard to the subcategories of “experienced”, PRs were lower when the cohabiting women had their experience as a health promotion volunteer longer ago, had experience in a leadership role, and who had higher satisfaction with their experience, although these differences were not statistically significant. When the cutoff value of functional capacity was changed from 10 points or less to 12 points or less, there was no remarkable change, though some of PRs showed a marginal significance (see Table S1).

Table 3 shows the association between the cohabiting women’s experience as a health promotion volunteer and depressive symptoms (GDS5) in the study population. Among those analyzed, 23.0% (532/2316) had depressive symptoms. The prevalence of depressive symptoms in the “experienced” group was lower than in the “not experienced” group (21.4% vs. 25.3%), and the PR was lower in Model 3 (adjusted PR; 0.84, 95% CI; 0.73–0.97). The PR was lowest when the cohabiting women had their experience as a health promotion volunteer “0–19 years” ago (0.80, 0.65–0.99), had experience in a leadership role (0.83, 0.64–1.08), and had those who had a “high” satisfaction with their experience (0.79, 0.62–1.01).

There were no remarkable changes in the results for both functional capacity and depressive symptoms, even when narrowing the age of cohabiting women to more than plus or minus 5 years old (see Table S2) and when not limiting the age of cohabiting women (see Table S3). In order to consider the effect of spousal concordance in depressive symptoms, we also conducted additional analysis which limited the health status of cohabiting women to not having depressive symptoms using their responses. As a result, adjusted PR of the “experienced” group was lower than in the “not experienced” group (0.86, 0.71–1.05), but not with statistical significance (see Table S4).

## 4. Discussion

In this study, cohabiting women’s experience as health promotion volunteers was inversely associated with depressive symptoms in elderly men from the same household, but not with men’s low functional capacity. In addition, the association was stronger when the women had their experience as a health promotion volunteer 0–19 years ago, had experience in a leadership role, and had a higher satisfaction with their experience.

Previous studies have shown that experience as a health promotion volunteer is associated with the volunteer’s own health conditions [6,7]. In this study, a similar association with health was also found in their male family members. In addition, previous studies have indicated that marital status has an effect on health. For example, mortality, frailty, and depression were at lower levels in men who had a spouse compared with men who had no spouse (not married, divorced, or widowed) [11,12,13,14]. In the current study, the sample was limited to married men, and the results suggested that there were differences of well-being even among married men, depending on the health promotion volunteer experience of the women they cohabited with. Further studies are needed to see whether other family members and neighbors are also affected by the women they associated with having experience as health promotion volunteer.

In the study, the cohabiting women’s experience as health promotion volunteers was associated with lower depressive symptoms, but not with the functional capacity, in elderly men from the same household. It has previously been shown that depressive symptoms in the elderly were associated with a future decline in ADL [25]. The results of our study may, therefore, cast light on the mechanism by which depressive symptoms were preceding the factor of impaired ADL. Cohabiting with women who have had experience as a health promotion volunteer may prevent future decline in functional capacity and ADL of the men from the same household by enabling them to maintain a good condition of mental well-being.

There are several possible mechanisms behind the positive effect on depressive symptoms. One hypothesis that explains the mechanism is improvement of health-related behaviors in elderly men. Health promotion volunteers are expected to make use of what they have learned in health learning programs to promote health, not only for themselves but also for their family or neighborhood (e.g., promotion of the exercise). However, the characteristics of the study population (Table 1) showed that there were no significant differences in health-related behaviors (exercise, healthy eating, drinking, and smoking). These results suggested that the experience as a health promotion volunteer might affect depressive symptoms in elderly men from the same household by other pathway. Another hypothesis is social support. Previous studies have suggested that social support from family, friends, or neighbors had a protective effect on mental health, including depressive symptoms [26,27,28,29,30]. Health promotion volunteers experience the activities that communicate with many people such as recommendations for health examination for their family and neighborhood. These experiences might enhance not only their health knowledge but also their support capabilities, particularly related to emotional support, which in turn could reduce stress in elderly men from the same household and have a positive effect on the depressive symptoms. In fact, GDS5 is composed of items such as life satisfaction and feeling of helplessness [22]. However, in our study, the specific activities taken part in as a health promotion volunteer were not measured. Therefore, this mechanism needs to be further investigated in order to see if it is correct.

In addition, depressive symptoms in elderly men might be affected by cohabiting women’s depressive symptoms itself. Previous studies have indicated that there is concordance in health status including depressive symptoms within married couples [15,16]. It might be possible that the cohabiting women’s experience as health promotion volunteers led to good condition of their own mental well-being, which in turn could bring the similar condition to their family as an intermediate variable. Therefore, we conducted additional analysis which limited the health status of cohabiting women to not having depressive symptoms using the responses of cohabiting women. The association turned to statistically nonsignificant, though the point estimates did not change remarkably (Table S4). However, because this study was a cross-sectional study, it is difficult to judge whether depressive symptoms of cohabiting women is the intermediate variable as described above or not (e.g., confounding variable). Further research such as longitudinal survey with larger sample size is needed in this regard.

We also examined the differences in the results depending on how long ago the women served as a health promotion volunteer, experience in a leadership role, and satisfaction with their experience. In terms of how long since the experience, the lowest adjusted PR for depressive symptoms was found in the category of “0–19 years”. This suggests that shorter the period since the served term as a health promotion volunteer, the more the experience might remain and be strongly associated with well-being of men from the same household. Further, in terms of experience in a leadership role, the lowest adjusted PR for depressive symptoms was found in in the “yes” group. It has previously been found that the numbers of days in which women took part in activities during their term were higher in the health promotion volunteers in a leadership position compared with those who were not [3]. This means that they had more opportunities to enhance their health knowledge and support capabilities and, therefore, the association with the well-being of men from the same household might be stronger. The women who reported a “high” satisfaction with their experience as health promotion volunteers were associated with the lowest adjusted PR for depressive symptoms. Satisfaction, which was based on self-evaluation, was considered an alternative indicator of how much the women were involved in the health promotion activities positively during their term, or how the activities had significant meaning for their life and health. Therefore, the association with the well-being of men from the same household might be stronger when the satisfaction was higher.

One of the advantages of this study was a relatively high response rate of the survey (77.7%), which can be considered to reflect the actual condition of the elderly people in Suzaka City well. In addition, our study considered not only medical history and health-related behaviors, but also socioeconomic status, including objective data on household income as covariates. There were, however, a number of limitations. First, there was a possibility of reverse causation. The results of this study might not indicate that the well-being of men was affected by the cohabiting women’s experience as a health promotion volunteer, but instead it may indicate that women in a household in which the well-being of cohabiting men may be elected as a health promotion volunteer more easily. However, health promotion volunteers in Suzaka City are elected mainly through a rotation system and serve 2-year terms [3]. Therefore, health promotion volunteers are elected almost at random and this experience as a health promotion volunteer should result in an enhancement of their health knowledge and social support capabilities, which leads to the high level of well-being in their family. However, we could not completely discount the possibility of reverse causation in the current study, so a further follow-up would be necessary to explore this. Second, the GDS5, which was used to determine depressive symptoms in this study, was not based on a clinical diagnosis but on a self-report of five question items. However, the validity of the GDS5 has previously been verified [23]. Third, as mentioned above, the specific activities that the health promotion volunteers engaged in were not measured in this study. Fourth, due to constraints on data collection, we were not able to identify married couples in the respondents. Therefore, the results of this study could not accurately indicate the health effect of the wife’s experience as health promotion volunteer to her husband. Finally, external validity might be an issue, since this study was conducted in a single region. Therefore, future research will be needed to see whether similar results can be found in other regions that have different health promotion volunteer activities or have different geographical conditions. Despite these limitations, this study provided evidence implying that the well-being of elderly men can be attributed to the activities of the women they cohabited with having experience as health promotion volunteers.

## 5. Conclusions

In conclusion, cohabiting women’s experience as a health promotion volunteer was inversely associated with depressive symptoms in elderly men from the same household, but not with functional capacity. In addition, the association were stronger when the women had volunteered 0–19 years ago, had experience in a leadership role, and had a higher satisfaction with the experience. These results give a suggestion to other regions (municipalities) that, in public health initiatives, elderly men may have better well-being by educating health promotion volunteers effectively.

## Figures and Tables

**Table 1:** Characteristics of the study population.

Variable	Experience as Health Promotion Volunteer of Cohabiting Woman				
	No *n* = 936		Yes *n* = 1434		*p*-value ^c^
Age (years)					
65–69	219	(23.4%)	237	(16.5%)	< 0.001
70–74	335	(35.8%)	482	(33.6%)	
75–79	203	(21.7%)	366	(25.5%)	
80–84	128	(13.7%)	242	(16.9%)	
≥85	51	(5.4%)	107	(7.5%)	
(Mean ± standard deviation years)	(74.0 ± 5.7)		(75.2 ± 5.8)		
Educational attainment (years)					
≥10	595	(63.6%)	961	(67.0%)	0.08
<10	341	(36.4%)	473	(33.0%)	
Equivalent household income ^a^					
1st quartile (–2.00 million yen)	124	(13.2%)	169	(11.8%)	0.06
2nd quartile (2.00–3.10 million yen)	257	(27.5%)	378	(26.4%)	
3rd quartile (3.10–4.24 million yen)	292	(31.2%)	410	(28.6%)	
4th quartile (4.24– million yen)	263	(28.1%)	477	(33.3%)	
History of major diseases ^b^					
No	506	(54.1%)	782	(54.5%)	0.82
Yes	430	(45.9%)	652	(45.5%)	
Exercise habits					
One hour or more per week	515	(55.0%)	794	(55.4%)	0.87
Less than one hour per week	421	(45.0%)	640	(44.6%)	
Consciousness of healthy eating habits					
Conscious	827	(88.4%)	1296	(90.4%)	0.12
Not conscious	109	(11.6%)	138	(9.6%)	
Current drinking					
No	333	(35.6%)	464	(32.4%)	0.11
Yes	603	(64.4%)	970	(67.6%)	
Current smoking					
No	782	(83.5%)	1220	(85.1%)	0.32
Yes	154	(16.5%)	214	(14.9%)	

^a^ Quartile in all respondents. ^b^ History of major diseases was defined as having any one of the following diseases: stroke, myocardial infarction/angina, diabetes, Parkinson’s disease, femoral neck fracture, and cancer. ^c^ Chi-square test.

**Table 2:** Association between cohabiting women’s experience as a health promotion volunteer and low functional capacity in the study population (*n* = 2247).

Experience as Health Promotion Volunteer of Cohabiting Woman	Outcome/Study Population (%)				Model 1 ^a^			Model 2 ^b^			Model 3 ^c^		
					PR (95% CI)^d^		*p*-Value	PR (95% CI)		*p*-Value	PR (95% CI)		*p*-Value
Presence of experience													
Not experienced	165	/	885	(18.6%)	1.00			1.00			1.00		
Experienced	249	/	1362	(18.3%)	0.94	(0.78–1.12)	0.46	0.97	(0.81–1.16)	0.74	1.02	(0.87–1.21)	0.79
Years since experience													
Not experienced	165	/	885	(18.6%)	1.00			1.00			1.00		
Experienced: 0–19 years	99	/	572	(17.3%)	1.01	(0.80–1.27)	0.93	1.07	(0.85–1.34)	0.57	1.12	(0.90–1.39)	0.30
: 20–39 years	121	/	639	(18.9%)	0.90	(0.73–1.12)	0.35	0.94	(0.76–1.16)	0.55	1.00	(0.82–1.23)	0.98
: 40 years or more	16	/	85	(18.8%)	0.75	(0.48–1.19)	0.22	0.78	(0.49–1.25)	0.30	0.86	(0.55–1.35)	0.52
: no response	13	/	66	(19.7%)	0.92	(0.55–1.52)	0.73	0.85	(0.52–1.38)	0.52	0.77	(0.50–1.21)	0.26
Leadership role													
Not experienced	165	/	885	(18.6%)	1.00			1.00			1.00		
Experienced: no	186	/	985	(18.9%)	0.98	(0.81–1.18)	0.84	1.01	(0.84–1.22)	0.92	1.06	(0.89–1.26)	0.54
: yes	40	/	267	(15.0%)	0.77	(0.56–1.06)	0.10	0.85	(0.62–1.16)	0.31	0.90	(0.67–1.22)	0.51
: no response	23	/	110	(20.9%)	0.94	(0.64–1.38)	0.74	0.90	(0.61–1.33)	0.59	0.98	(0.68–1.42)	0.93
Satisfaction with the experience													
Not experienced	165	/	885	(18.6%)	1.00			1.00			1.00		
Experienced: low	37	/	152	(24.3%)	1.30	(0.96–1.77)	0.10	1.29	(0.96–1.75)	0.09	1.27	(0.95–1.70)	0.11
: medium	161	/	842	(19.1%)	0.98	(0.80–1.19)	0.83	1.02	(0.84–1.24)	0.84	1.08	(0.90–1.30)	0.39
: high	43	/	311	(13.8%)	0.70	(0.51–0.95)	0.02	0.74	(0.54–1.00)	0.05	0.81	(0.60–1.10)	0.18
: no response	8	/	57	(14.0%)	0.68	(0.35–1.30)	0.24	0.65	(0.34–1.24)	0.19	0.62	(0.35–1.10)	0.10

PR, prevalence ratio; CI, confidence interval. ^a^ Model 1. Adjusted for age (continuous). ^b^ Model 2. Adjusted for age (continuous), educational attainment, and equivalent household income. ^c^ Model 3. Adjusted for age (continuous), educational attainment, equivalent household income, history of major diseases, exercise habits, consciousness of healthy eating habits, current drinking, and current smoking. ^d^ Adjusted PR and 95% CI were estimated by modified Poisson regression analysis.

**Table 3:** Association between cohabiting women’s experience as a health promotion volunteer and depressive symptoms in the study population (*n* = 2316).

Experience as Health Promotion Volunteer of Cohabiting Woman	Outcome/Study Population (%)				Model 1 ^a^			Model 2 ^b^			Model 3 ^c^		
					PR (95% CI) ^d^		*p*-Value	PR (95% CI)		*p*-Value	PR (95% CI)		*p*-Value
Presence of experience													
Not experienced	232	/	917	(25.3%)	1.00			1.00			1.00		
Experienced	300	/	1399	(21.4%)	0.81	(0.70–0.94)	0.01	0.82	(0.71–0.95)	0.01	0.84	(0.73–0.97)	0.02
Years since experience													
Not experienced	232	/	917	(25.3%)	1.00			1.00			1.00		
Experienced: 0–19 years	104	/	579	(18.0%)	0.77	(0.62–0.95)	0.01	0.78	(0.63–0.96)	0.02	0.80	(0.65–0.99)	0.04
: 20–39 years	157	/	660	(23.8%)	0.85	(0.71–1.01)	0.07	0.86	(0.72–1.03)	0.10	0.89	(0.75–1.06)	0.20
: 40 years or more	26	/	94	(27.7%)	0.83	(0.58–1.18)	0.30	0.84	(0.59–1.21)	0.36	0.87	(0.61–1.24)	0.46
: no response	13	/	66	(19.7%)	0.70	(0.43–1.14)	0.16	0.67	(0.41–1.09)	0.11	0.64	(0.40–1.02)	0.06
Leadership role													
Not experienced	232	/	917	(25.3%)	1.00			1.00			1.00		
Experienced: no	218	/	1,008	(21.6%)	0.83	(0.71–0.97)	0.02	0.84	(0.72–0.99)	0.03	0.86	(0.73–1.00)	0.05
: yes	55	/	271	(20.3%)	0.77	(0.59–0.99)	0.05	0.79	(0.61–1.03)	0.08	0.83	(0.64–1.08)	0.16
: no response	27	/	120	(22.5%)	0.74	(0.52–1.03)	0.08	0.72	(0.51–1.02)	0.06	0.76	(0.54–1.06)	0.11
Satisfaction with the experience													
Not experienced	232	/	917	(25.3%)	1.00			1.00			1.00		
Experienced: low	35	/	160	(21.9%)	0.85	(0.63–1.16)	0.31	0.85	(0.63–1.16)	0.31	0.83	(0.62–1.11)	0.20
: medium	189	/	858	(22.0%)	0.83	(0.70–0.98)	0.03	0.84	(0.71–1.00)	0.05	0.88	(0.74–1.03)	0.12
: high	65	/	324	(20.1%)	0.74	(0.58–0.95)	0.02	0.76	(0.60–0.97)	0.03	0.79	(0.62–1.01)	0.06
: no response	11	/	57	(19.3%)	0.70	(0.41–1.20)	0.20	0.68	(0.40–1.16)	0.16	0.68	(0.40–1.13)	0.13

PR, prevalence ratio; CI, confidence interval. ^a^ Model 1. Adjusted for age (continuous). ^b^ Model 2. Adjusted for age (continuous), educational attainment, and equivalent household income. ^c^ Model 3. Adjusted for age (continuous), educational attainment, equivalent household income, history of major diseases, exercise habits, consciousness of healthy eating habits, current drinking, and current smoking. ^d^ Adjusted PR and 95% CI were estimated by modified Poisson regression analysis.

## Supplementary Materials

The following are available online at http://www.mdpi.com/1660-4601/16/1/65/s1, Table S1: Association between cohabiting women’s experience as a health promotion volunteer and low functional capacity (12 points or less) in the study population, Table S2: Associations between cohabiting women’s experience as a health promotion volunteer and both outcomes in the study population when narrowing the age of cohabiting women, Table S3: Associations between cohabiting women’s experience as a health promotion volunteer and both outcomes in the study population when not limiting the age of cohabiting women, Table S4: Associations between cohabiting women’s experience as a health promotion volunteer and depressive symptoms in the study population when limiting the health status of cohabiting women to not having depressive symptoms.
