# Supplementary material for: Relationship between the Well-Being of Elderly Men and Cohabiting with Women Who Have Had Experience as a Health Promotion Volunteer in Japan: A Cross-Sectional Study
Source: Int J Environ Res Public Health. 2018 Dec 27;16(1):65. doi: 10.3390/ijerph16010065 (PMC6338992; doi:10.3390/ijerph16010065)
Supplement: Supplementary file 1 [file ijerph-16-00065-s001.pdf]

Supplementary Material

Relationship between the Well-Being of Elderly Men and Cohabiting with Women Who Have Had Experience as a Health Promotion Volunteer in Japan: A Cross-Sectional Study

Haruhiko Imamura [1,*], Hideki Nakamura [2] and Yuji Nishiwaki [1]

Table S1. Association between cohabiting women's experience as a health promotion volunteer and low functional capacity (12 points or less) in the study population (n = 2247).

Experience as Health Promotion Volunteer of Cohabiting Woman	Outcome / Study Population (%)	Model 1 [a]		Model 2 [b]		Model 3 [c]	
		PR (95% CI) [d]	*p*-Value	PR (95% CI)	*p*-Value	PR (95% CI)	*p*-Value
**Presence of experience**							
Not experienced	457 / 885 (51.6%)	1.00		1.00		1.00	
Experienced	656 / 1362 (48.2%)	0.91 (0.84 – 0.99)	0.04	0.92 (0.85 – 1.01)	0.07	0.94 (0.87 – 1.02)	0.14
**Years since experience**							
Not experienced	457 / 885 (51.6%)	1.00		1.00		1.00	
Experienced: 0–19 years	268 / 572 (46.9%)	0.94 (0.84 – 1.04)	0.23	0.95 (0.85 – 1.06)	0.37	0.97 (0.87 – 1.07)	0.52
: 20–39 years	301 / 639 (47.1%)	0.87 (0.78 – 0.97)	0.01	0.88 (0.79 – 0.98)	0.02	0.90 (0.81 – 1.00)	0.04
: 40 years or more	52 / 85 (61.2%)	1.05 (0.87 – 1.27)	0.58	1.07 (0.88 – 1.29)	0.50	1.09 (0.90 – 1.31)	0.37
: no response	35 / 66 (53.0%)	0.97 (0.77 – 1.23)	0.83	0.95 (0.75 – 1.19)	0.64	0.92 (0.74 – 1.16)	0.50
**Leadership role**							
Not experienced	457 / 885 (51.6%)	1.00		1.00		1.00	
Experienced: no	474 / 985 (48.1%)	0.92 (0.84 – 1.01)	0.07	0.93 (0.85 – 1.01)	0.10	0.94 (0.86 – 1.03)	0.17
: yes	128 / 267 (47.9%)	0.91 (0.79 – 1.05)	0.19	0.94 (0.82 – 1.08)	0.41	0.97 (0.84 – 1.11)	0.62
: no response	54 / 110 (49.1%)	0.88 (0.72 – 1.08)	0.21	0.86 (0.71 – 1.05)	0.15	0.89 (0.73 – 1.08)	0.23
**Satisfaction with the experience**							
Not experienced	457 / 885 (51.6%)	1.00		1.00		1.00	
Experienced: low	80 / 152 (52.6%)	1.02 (0.86 – 1.20)	0.84	1.02 (0.87 – 1.19)	0.85	1.00 (0.86 – 1.17)	0.97
: medium	413 / 842 (49.0%)	0.93 (0.85 – 1.02)	0.13	0.94 (0.86 – 1.04)	0.22	0.96 (0.88 – 1.05)	0.39
: high	138 / 311 (44.4%)	0.84 (0.73 – 0.96)	0.01	0.85 (0.74 – 0.98)	0.03	0.88 (0.76 – 1.01)	0.06

: no response	25	/	57	(43.9%)	0.81	(0.60	–	1.10)	0.18	0.80	(0.60	–	1.07)	0.14	0.80	(0.60	–	1.07)	0.13

PR, prevalence ratio; CI, confidence interval. * This table shows the results for low functional capacity when the cutoff value was changed to 12 points or less. [a] Model 1. Adjusted for age (continuous). [b] Model 2. Adjusted for age (continuous), educational attainment, and equivalent household income. [c] Model 3. Adjusted for age (continuous), educational attainment, equivalent household income, history of major diseases, exercise habits, consciousness of healthy eating habits, current drinking, and current smoking. [d] Adjusted PR and 95% CI were estimated by modified Poisson regression analysis.

Table S2. Associations between cohabiting women's experience as a health promotion volunteer and both outcomes in the study population when narrowing the age of cohabiting women.

Experience as Health Promotion Volunteer of Cohabiting Woman	[Model 3 for Outcome 1] [a] Low Functional Capacity (n = 1928)					[Model 3 for Outcome 2] Depressive Symptoms (n = 1986)				
	PR (95% CI) [b]				p-Value	PR (95% CI)				p-Value
**Presence of experience**										
Not experienced	1.00					1.00				
Experienced	1.03	(0.86	–	1.23)	0.75	0.86	(0.74	–	1.01)	0.06
**Years since experience**										
Not experienced	1.00					1.00				
Experienced: 0–19 years	1.13	(0.89	–	1.42)	0.31	0.82	(0.66	–	1.02)	0.08
: 20–39 years	1.02	(0.82	–	1.26)	0.89	0.92	(0.76	–	1.11)	0.37
: 40 years or more	0.71	(0.40	–	1.23)	0.22	0.83	(0.55	–	1.28)	0.41
: no response	0.87	(0.57	–	1.35)	0.54	0.73	(0.47	–	1.15)	0.18
**Leadership role**										
Not experienced	1.00					1.00				
Experienced: no	1.07	(0.89	–	1.29)	0.49	0.86	(0.72	–	1.02)	0.08
: yes	0.95	(0.68	–	1.33)	0.76	0.92	(0.69	–	1.21)	0.54
: no response	0.86	(0.57	–	1.31)	0.48	0.78	(0.54	–	1.15)	0.21
**Satisfaction with the experience**										
Not experienced	1.00					1.00				
Experienced: low	1.31	(0.97	–	1.77)	0.08	0.79	(0.57	–	1.10)	0.17
: medium	1.08	(0.89	–	1.31)	0.45	0.89	(0.75	–	1.07)	0.22
: high	0.81	(0.59	–	1.12)	0.21	0.83	(0.64	–	1.07)	0.15
: no response	0.73	(0.42	–	1.27)	0.27	0.80	(0.49	–	1.31)	0.37

PR, prevalence ratio; CI, confidence interval. * This table shows the results for low functional capacity and depressive symptoms when narrowing the age of cohabiting women to more than plus or minus 5 years old in Model 3. [a] Model 3. Adjusted for age (continuous), educational attainment, equivalent household income, history of major diseases, exercise habits, consciousness of healthy eating habits, current drinking, and current smoking. [b] Adjusted PR and 95% CI were estimated by modified Poisson regression analysis.

Table S3. Associations between cohabiting women's experience as a health promotion volunteer and both outcomes in the study population when not limiting the age of cohabiting women.

Experience as Health Promotion Volunteer of Cohabiting Woman	[Model 3 for Outcome 1] [a] Low Functional Capacity (n = 2261)					[Model 3 for Outcome 2] Depressive Symptoms (n = 2330)				
	PR (95% CI) [b]				p-Value	PR (95% CI)				p-Value
**Presence of experience**										
Not experienced	1.00					1.00				
Experienced	1.03	(0.87	–	1.22)	0.72	0.85	(0.74	–	0.98)	0.03
**Years since experience**										
Not experienced	1.00					1.00				
Experienced: 0–19 years	1.13	(0.91	–	1.40)	0.28	0.82	(0.67	–	1.00)	0.05
: 20–39 years	1.01	(0.83	–	1.24)	0.90	0.90	(0.76	–	1.07)	0.23
: 40 years or more	0.87	(0.56	–	1.36)	0.54	0.87	(0.61	–	1.24)	0.44
: no response	0.78	(0.50	–	1.22)	0.27	0.64	(0.40	–	1.01)	0.06
**Leadership role**										
Not experienced	1.00					1.00				
Experienced: no	1.07	(0.89	–	1.27)	0.48	0.86	(0.74	–	1.01)	0.06
: yes	0.91	(0.67	–	1.23)	0.54	0.85	(0.66	–	1.09)	0.20
: no response	0.99	(0.69	–	1.42)	0.95	0.75	(0.54	–	1.06)	0.10
**Satisfaction with the experience**										
Not experienced	1.00					1.00				
Experienced: low	1.33	(1.00	–	1.77)	0.05	0.86	(0.65	–	1.15)	0.31
: medium	1.08	(0.90	–	1.30)	0.39	0.88	(0.75	–	1.04)	0.14
: high	0.81	(0.60	–	1.10)	0.18	0.79	(0.62	–	1.00)	0.06
: no response	0.62	(0.35	–	1.11)	0.11	0.68	(0.41	–	1.13)	0.14

PR, prevalence ratio; CI, confidence interval. * This table shows the results for low functional capacity and depressive symptoms when not limiting the age of cohabiting women in Model 3. [a] Model 3. Adjusted for age (continuous), educational attainment, equivalent household income, history of major diseases, exercise habits, consciousness of healthy eating habits, current drinking, and current smoking. [b] Adjusted PR and 95% CI were estimated by modified Poisson regression analysis.

Table S4. Associations between cohabiting women's experience as a health promotion volunteer and depressive symptoms in the study population when limiting the health status of cohabiting women to not having depressive symptoms (n = 1670).

Experience as Health Promotion Volunteer of Cohabiting Woman	Outcome / Study Population (%)				Model 3 [a]				
					PR (95% CI)				p-Value
**Presence of experience**									
Not experienced	122	/	629	(19.4%)	1.00				
Experienced	183	/	1041	(17.6%)	0.86	(0.71	–	1.05)	0.15
**Years since experience**									
Not experienced	122	/	629	(19.4%)	1.00				
Experienced: 0–19 years	74	/	459	(16.1%)	0.88	(0.68	–	1.14)	0.33
: 20–39 years	90	/	485	(18.6%)	0.87	(0.68	–	1.10)	0.24
: 40 years or more	14	/	64	(21.9%)	0.90	(0.54	–	1.51)	0.69
: no response	5	/	33	(15.2%)	0.60	(0.31	–	1.18)	0.14
**Leadership role**									
Not experienced	122	/	629	(19.4%)	1.00				
Experienced: no	132	/	749	(17.6%)	0.86	(0.70	–	1.07)	0.18
: yes	40	/	230	(17.4%)	0.89	(0.64	–	1.23)	0.47
: no response	11	/	62	(17.7%)	0.80	(0.46	–	1.37)	0.41
**Satisfaction with the experience**									
Not experienced	122	/	629	(19.4%)	1.00				
Experienced: low	17	/	101	(16.8%)	0.77	(0.50	–	1.19)	0.23
: medium	118	/	652	(18.1%)	0.89	(0.72	–	1.12)	0.32
: high	44	/	260	(16.9%)	0.86	(0.63	–	1.16)	0.32
: no response	4	/	28	(14.3%)	0.64	(0.30	–	1.39)	0.26

PR, prevalence ratio; CI, confidence interval. * This table shows the results for depressive symptoms when limiting the health status of cohabiting women to not having depressive symptoms in Model 3. [a] Model 3. Adjusted for age (continuous), educational attainment, equivalent household income, history of major diseases, exercise habits, consciousness of healthy eating habits, current drinking, and current smoking. [b] Adjusted PR and 95% CI were estimated by modified Poisson regression analysis.

© 2018 by the authors. Submitted for possible open access publication under the terms and conditions of the Creative Commons Attribution (CC BY) license (http://creativecommons.org/licenses/by/4.0/).
